# Correction to: Rapid assessment of changes in phage bioactivity using dynamic light scattering

**DOI:** 10.1093/pnasnexus/pgae136

**Published:** 2024-04-03

This is a correction to: Tejas Dharmaraj *et al.*, Rapid assessment of changes in phage bioactivity using dynamic light scattering, *PNAS Nexus*, Volume 2, Issue 12, December 2023, pgad406, https://doi.org/10.1093/pnasnexus/pgad406

In the originally published version, the statement of competing interests indicated that A.O.-B., J.D.v.B., and R.M. are members of Felix Biotechnology, Inc., a phage therapy company.

This has been corrected to read: A.O.-B., J.D.B., and R.M. are members of Felix Biotechnology, Inc., a phage therapy company.

