# Supplementary material for: Rapid assessment of changes in phage bioactivity using dynamic light scattering
Source: PNAS Nexus. 2023 Nov 27;2(12):pgad406. doi: 10.1093/pnasnexus/pgad406 (PMC10726995; doi:10.1093/pnasnexus/pgad406)
Supplement: pgad406_Supplementary_Data [file pgad406_supplementary_data.zip › PNASNEXUS-PNASNEXUS-2023-00539R-s01.docx]

**
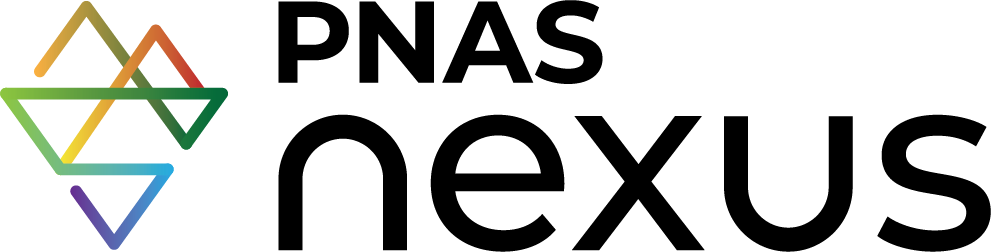
**

**Supplementary Information for**

Rapid assessment of changes in phage bioactivity using dynamic light scattering

Tejas Dharmaraj^1,2^, Michael J. Kratochvil^3^, Julie D. Pourtois^4^, Qingquan Chen^1^, Maryam Hajfathalian^1^, Aviv Hargil^1^, Yung-Hao Lin^5^, Zoe Evans^1^, Agnès Oromí-Bosch^6^, Joel D. Berry^6^, Robert McBride^6^, Naomi L. Haddock^1^, Derek R. Holman^7^, Jonas D. van Belleghem^1^, Tony H. Chang^1^, Jeremy J. Barr^8^, Rob Lavigne^9^, Sarah C. Heilshorn^3^, Francis G. Blankenberg^10^, Paul L. Bollyky^1^.

^1^Division of Infectious Diseases and Geographic Medicine, Department of Medicine, Stanford University School of Medicine, Stanford, CA 94305, USA.

^2^Sarafan ChEM-H, Stanford University, Stanford, CA 94305, USA.

^3^Department of Materials Science and Engineering, Stanford University, Stanford, CA 94305

^4^Hopkins Marine Station, Department of Biology, Stanford University, Pacific Grove, CA 93950, USA.

^5^Department of Chemical Engineering, Stanford University, Stanford, CA 94305, USA.

^6^Felix Biotechnology, South San Francisco, CA, 94080.

^7^Division of Gastroenterology and Hepatology, Department of Medicine, Stanford University School of Medicine, Stanford, CA 94305, USA.

^8^School of Biological Sciences, Monash University, Clayton, 3800, VIC, Australia

^9^Department of Biosystems, KU Leuven, Leuven, 3001, Belgium

^10^Division of Pediatric Radiology and Nuclear Medicine, Department of Radiology, Lucile Packard Children’s Hospital, Stanford, CA 94305, USA.

**Corresponding Author:** Paul L. Bollyky, MD, PhD, Division of Infectious Diseases and Geographic Medicine, Department of Medicine, Stanford University School of Medicine, Beckman Center for Molecular and Genetic Medicine, 279 Campus Drive, Stanford, CA 94305, USA. pbollyky@stanford.edu.

**Email:**  pbollyky@stanford.edu

**This PDF file includes:**

Figures S1 to S3

Tables S1 to S2

**Other supplementary materials for this manuscript include:**

Dataset S1


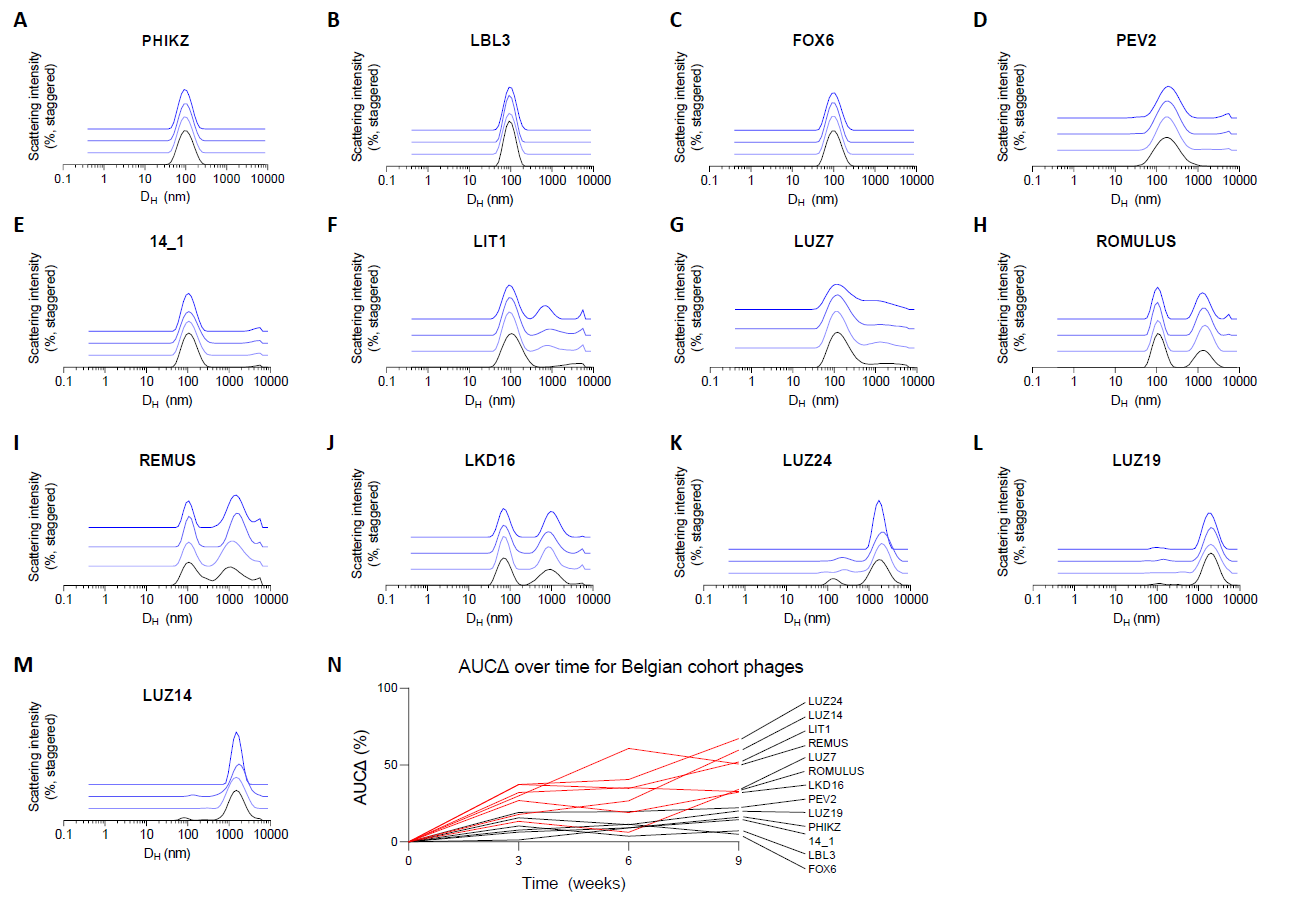


**Fig. S1. Phages spontaneously fragment and aggregate in refrigerated storage over time (Belgian cohort).**

(**A** through **M**) DLS spectra of phages from the Belgian cohort (BC) over a monitoring period of two months. Shown are averages of n=3 DLS measurements per phage. n=13 phages were assessed. Darker colors and vertical staggering are used to show progression in time. Few phages remained largely intact (**A** through **C**). Most phages aggregated over the monitoring period (**D** through **M**). (**N**) AUCΔ identifies the most-changed phage over the monitoring period and is consistent with our qualitative assessment of the DLS spectra.


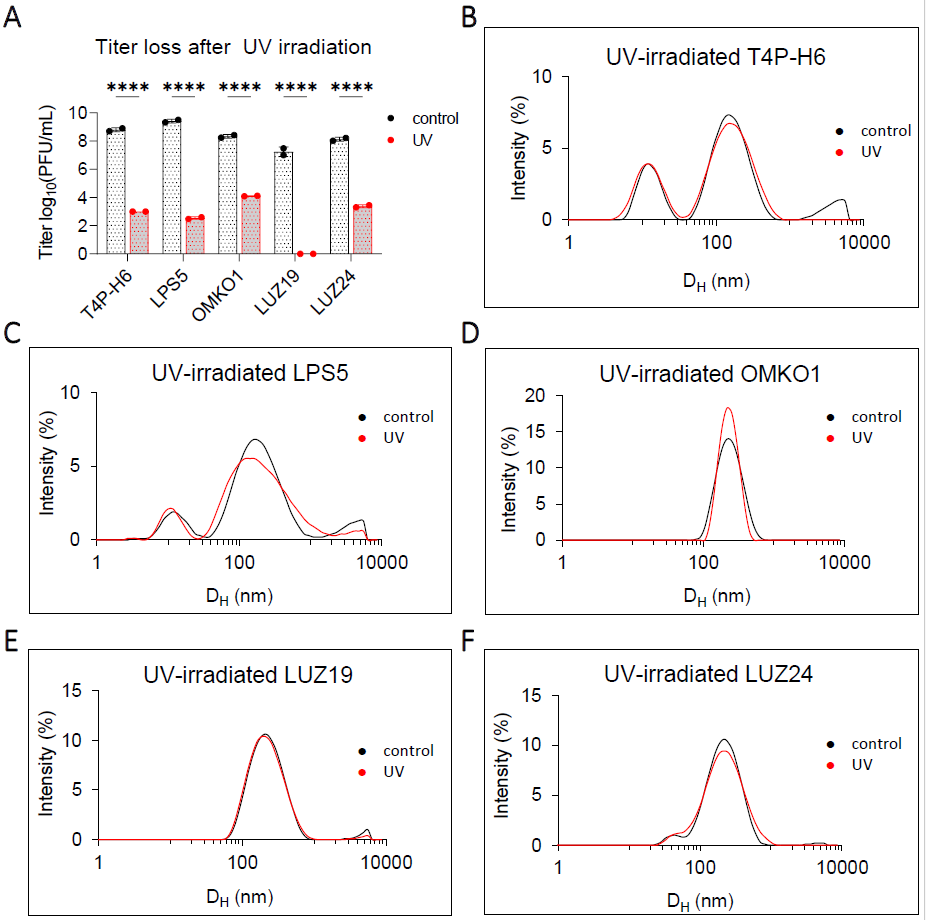


**Fig. S2: DLS captures changes in phage size, but it does not capture genomic damage.**

(**A**) Titer of phages after irradiation for 20 minutes with germicidal UV-C light. Results are from one experiment. Titer was measured with n=2 plaque assays per phage per condition. Two-way ANOVA with Tukey correction. **** = p < 0.0001. (**B** through **F**) DLS spectra of phages before and after irradiation. Shown are averages of n=3 DLS measurements per phage per condition.


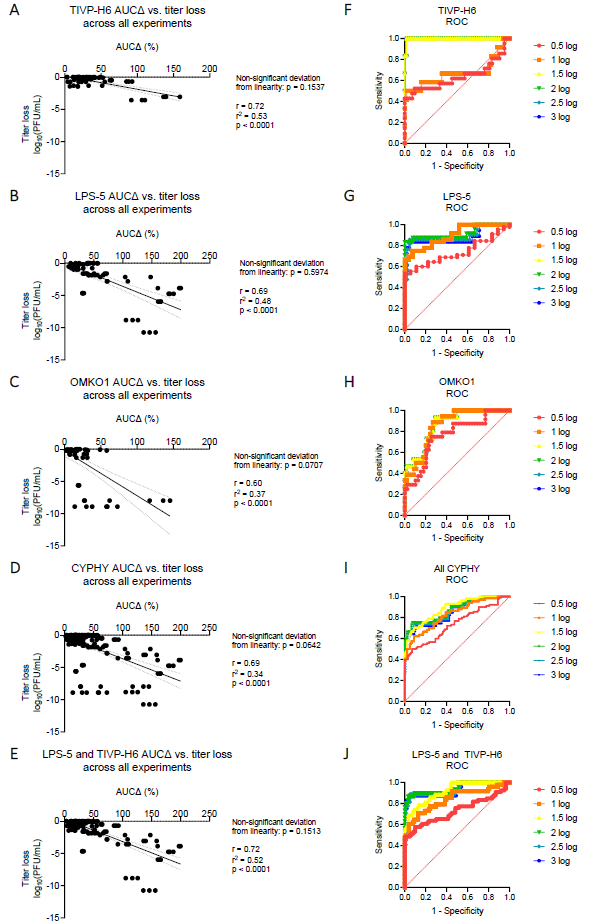


**Fig. S3: Negative linear associations between AUCΔ and titer loss, and ROC of logistic models.**

(**A** through **E**) AUCΔ vs. titer loss and (**F** through **J**) Receiver Operating Characteristic (ROC) for (**A** and **F**) TIVP-H6, (**B** and **G**) LPS-5, (**C** and **H**) OMKO1, all CYPHY phages (**D** and **I**), and (**E** and **J**) LPS-5 and TIVP-H6 together. Runs test for significant deviations from linearity. Two-tailed Pearson’s test for significance of correlation.

**Table S1. Physical and biological characteristics of CYPHY phages.**

| **Phage** | **TIVP-H6** | **LPS-5** | **OMKO1** |
| --- | --- | --- | --- |
| **Plaque characteristics** | Clear, well-circumscribed  Size: 0.3 – 1 mm | Clear, well-circumscribed  Size: 1.5 – 2.0 mm | Clear, well-circumscribed  Size: 0.5 – 1.5 mm |
| **Dimensions** | Tail length: 190 ± 5 nm  Tail width: 16 ± 2 nm  Head length: 74 ± 2 nm  Head shape: isometric | Tail length: 140 ± 2 nm  Tail width: 20 ± 1 nm  Head length: 70 ± 3 nm  Head shape: isometric | Tail length: 212 ± 2 nm  Tail width: 28 ± 1 nm  Head length: 134 ± 4 nm  Head shape: isometric |
| **Morphology** | *Siphoviridae* | *Myoviridae* | *Myoviridae* |

**Table S2. Phages used in this study.**

| **Phage** | **Taxonomy** | **Bacterial Host** | **Morphology** | **genome size (kb)** | **dim. (nm)**  **capsid / tail** |
| --- | --- | --- | --- | --- | --- |
| LUZ14 | *Autographiviridae* | *P. aeruginosa* C1 | podovirus | ~43 | 62/12 |
| LUZ19 | *Autographiviridae, Phikmvvirus* | *P. aeruginosa* PA01 K | podovirus | 43.5 | 65/12 |
| LUZ24 | *Bruynoghevirus* | *P. aeruginosa* Li010 | podovirus | 45.6 | 63/12 |
| LKD16 | *Autographiviridae, Phikmvvirus* | *P. aeruginosa* GHB15 | podovirus | 43.2 | 65/12 |
| LUZ7 | *Schitoviridae, Luzseptimavirus* | *P. aeruginosa* Br257 | podovirus | 74.9 | 76/30 |
| PEV2 | *Schitoviridae, Litunavirus* | *P. aeruginosa* PA01 K | podovirus | 72.7 | 70/30 |
| LIT1 | *Schitoviridae, Litunavirus* | *P. aeruginosa* US449 | myovirus | 72.5 | 74/30 |
| LBL3 | *Pbunavirus* | *P. aeruginosa* C1 | myovirus | 64.4 | 73/148 |
| 14_1 | *Pbunavirus* | *P. aeruginosa* Li010 | myovirus | 66.2 | 73/148 |
| PhiKZ | *Phikzvirus* | *P. aeruginosa* Aa245 | myovirus | 280.3 | 145/200 |
| Romulus | *Herelleviridae, Silviavirus* | *S. aureus* (broad host range) | myovirus | 131.3 | 90/204 |
| Remus | *Herelleviridae, Silviavirus* | *S. aureus* (broad host range) | myovirus | 134.6 | 90/204 |
| Fox6 | *Carmasinavirus* | *X. campestris* pv. *campestris* I11008 | myovirus | 61.1 | 78/156 |
| OMKO1 | *Phikzvirus* | *P. aeruginosa* PAO1 | myovirus | 281.8 | 134/212 |
| LPS-5 | *Pakpunavirus* | *P. aeruginosa* PAO1 | myovirus | 93.1 | 70/140 |
| TIVP-H6 | *Nipunavirus* | *P. aeruginosa* PA14 | siphovirus | 57.4 | 74/190 |
| T2 | *Straboviridae, Tequatrovirus* | *E. coli* B | myovirus | 163.8 | 111/78 |
| T3 | *Autographiviridae, Teetrevirus* | *E. coli* B | podovirus | 38.3 | 60/30 |
| T4 | *Straboviridae, Tequatrovirus* | *E. coli* B | myovirus | 168.9 | 111/78 |
| T6 | *Straboviridae, Tequatrovirus* | *E. coli* B | myovirus | 170 | 120/86 |
| T7 | *Autographiviridae, Tespetimavirus* | *E. coli* B | podovirus | 39.9 | 55/29 |
